# Pituitary Extract

**Published:** 1912-09

**Authors:** 


Pituitary Extract.—Of the various organo-therapeutic agents which have been carefully studied and brought into prominence during recent years, none is more interesting than the infundibular extract of the substance of the pituitary gland.

Having a wide range of usefulness, this agent promises to become an important addition to those measures which have been used more or less successfully in various obscure conditions.

In an attempt to solve the problem of overcoming the temporary paralysis due to exposure of the intestines following laparotomies, Birdwell (*Clin. Journal*, September, 1911), tried the injection of pituitary extract in a series of twenty uncomplicated cases. He found the results gratifying, the drug having had a marked effect on the muscular coats of the intestines. None of the patients so treated complained of flatulence and the early passage of flatus in each case was a marked feature, as a rule. Doses of 0.5 to 1 c.c. were injected intramuscularly, at intervals of six hours, for one to three days, until results were obtained.

It has been experimentally proven that pituitary extract not only stimulates the muscles of the bladder and uterus, but also the independent nerve supply of these organs. Hoffstatter (Wien Klin-Woch. 49, 1911) was able to overcome post-operative atony of the bladder in a large number of gynecological cases, by the intramuscular injection of from ½ c.c. pituitrin, thereby doing away with the use of the catheter. Thus he added much comfort and safety to the patients. Each injection was followed by voluntary urination in from fifteen minutes to an hour.

In obstetrical practice, pituitary extract has found favor with many. It may be tried, both in the induction of delayed labor and as an adjunct to ergot in post-partum atony of the uterus and post-partum hemorrhage. It would seem, however, that the drug is much more effective in inducing contractions of the uterus at full-term than during the earlier months of pregnancy. In a case observed recently by the writer, a primipara, twelve days overdue, was given an intramuscular injection of 1 c.c. of pituitrin without effect; five hours later, 2 c.c. of pituitrin similarly injected was soon followed by good contractions of the uterus which continued throughout labor. In this case, the introduction of hydrostatic bags to induce labor was avoided.

Aside from the uses described above, pituitary extract, on account of its property to raise and maintain blood pressure over a considerable period, may be used with advantage in shock and collapse. It is also said to increase greatly the flow of urine and may thus be employed in cases of anuria, providing the blood pressure of the patient is not high. The extract of the pituitary gland appears on the market as pituitrin and as pituitary (infundibular) extract. The average stated dose is 0.5 to 1 c.c., given preferably by the intramuscular method.—*Medical Review of Reviews.*

---

After a transpleural intrathoracic operation, as on the esophagus or lung, air-tight drainage of the pleural cavity must be provided for.—*American Journal of Surgery.*

On July 30, President Taft, with the confirmation of the Senate, commissioned Dr. William Edward Fitch, editor of *Pediatrics,* of New York City, to be a first lieutenant in the Medical Reserve Corps of the Army of the United States, dating from July 3, 1912.—*Va. Semi-Monthly.*

